# Association study of brain structure–function coupling and glymphatic system function in patients with mild cognitive impairment due to Alzheimer’s disease

**DOI:** 10.3389/fnins.2024.1417986

**Published:** 2024-07-29

**Authors:** Yong-Wen Sun, Xin-Yue Lyu, Xiao-Yang Lei, Ming-Ming Huang, Zhen-Min Wang, Bo Gao

**Affiliations:** ^1^Department of Radiology, The Affiliated Hospital of Guizhou Medical University, Guiyang, China; ^2^Department of Neurology, The Affiliated Hospital of Guizhou Medical University, Guiyang, China; ^3^Key Laboratory of Brain Imaging, Guizhou Medical University, Guiyang, China

**Keywords:** glymphatic system, DTI-ALPS, functional connectivity, mild cognitive impairment, magnetic resonance imaging

## Abstract

**Background:**

Mild cognitive impairment (MCI) is a critical transitional phase from healthy cognitive aging to dementia, offering a unique opportunity for early intervention. However, few studies focus on the correlation of brain structure and functional activity in patients with MCI due to Alzheimer’s disease (AD). Elucidating the complex interactions between structural-functional (SC-FC) brain connectivity and glymphatic system function is crucial for understanding this condition.

**Method:**

The aims of this study were to explore the relationship among SC-FC coupling values, glymphatic system function and cognitive function. 23 MCI patients and 18 healthy controls (HC) underwent diffusion tensor imaging (DTI) and resting-state functional MRI (fMRI). DTI analysis along the perivascular space (DTI-ALPS) index and SC-FC coupling values were calculated using DTI and fMRI. Correlation analysis was conducted to assess the relationship between Mini-Mental State Examination (MMSE) scores, DTI-ALPS index, and coupling values. Receiver operating characteristic (ROC) curves was conducted on the SC-FC coupling between the whole brain and subnetworks. The correlation of coupling values with MMSE scores was also analyzed.

**Result:**

MCI patients (67.74 ± 6.99 years of age) exhibited significantly lower coupling in the whole-brain network and subnetworks, such as the somatomotor network (SMN) and ventral attention network (VAN), than HCs (63.44 ± 6.92 years of age). Whole-brain network coupling was positively correlated with dorsal attention network (DAN), SMN, and visual network (VN) coupling. MMSE scores were significantly positively correlated with whole-brain coupling and SMN coupling. In MCI, whole-brain network demonstrated the highest performance, followed by the SMN and VAN, with the VN, DAN, limbic network (LN), frontoparietal network (FPN), and default mode network (DMN). Compared to HCs, lower DTI-ALPS index was observed in individuals with MCI. Additionally, the left DTI-ALPS index showed a significant positive correlation with MMSE scores and coupling values in the whole-brain network and SMN.

**Conclusion:**

These findings reveal the critical role of SC-FC coupling values and the ALPS index in cognitive function of MCI. The positive correlations observed in the left DTI-ALPS and whole-brain and SMN coupling values provide a new insight for investigating the asymmetrical nature of cognitive impairments.

## 1 Introduction

Alzheimer’s disease (AD) is a progressive, degenerative disorder affecting the central nervous system that is typically characterized by cognitive decline, abnormal behavior, and diminished daily living skills (Alzheimer's Association Report, 2023). Mild cognitive impairment (MCI) is an objective cognitive decline that can be confirmed by neuropsychological tests and is characterized by memory loss that is not consistent with individuals of the same age. The patient’s ability to perform complex daily tasks is mildly impaired, and basic daily living is unaffected, but not enough for a diagnosis of dementia (Busse et al., 2006). MCI represents a transitional phase between normal aging and dementia, with individuals being at a high risk of developing AD dementia. As there are currently no treatments that can fundamentally alter the course of AD, early diagnosis is crucial (Lane et al., 2018).

In this context, the roles of diffusion tensor imaging (DTI) and resting-state functional MRI (fMRI) are paramount. DTI explores the structural connectivity in the brain, while fMRI assesses brain functional connectivity (Wang et al., 2015; Teipel et al., 2016; Zhou et al., 2017). The analysis of structural and functional connectivity combined, also known as “structure–function coupling” (SC-FC), can elucidate the intricate relationship between brain anatomy and functional activity. Investigating this relationship is vital for understanding how the brain orchestrates complex cognitive tasks (Honey et al., 2009). Although functional brain connectivity in a resting state varies and often occurs between regions not directly connected anatomically, it is still constrained by the macroscopic anatomy of the cerebral cortex. Functional connectivity can indirectly shape structural connectivity, influencing the refinement of structural networks (Hagmann et al., 2010; Guerra-Carrillo et al., 2014). This ability highlights the role of white matter maturation in the restructuring of these networks during late brain development and underscores the importance of combining resting-state fMRI and DTI as tools for observing brain plasticity (Gatto, 2020).

The study of the relationship between structural connectivity (SC) and functional connectivity (FC) originates from neuroscience and aims to decipher how the brain network’s topological structure influences network dynamics and, in turn, affects human cognitive functions. Research on SC or FC in neuroscience primarily concentrates on cortical areas, revealing differences in human brain development, aging, and between healthy conditions and neuropsychiatric disorders. At the neuronal level, this research has unveiled the existence of scale-free topological structures, characterized by hub neurons, characteristic motifs, and dense clusters (Honey et al., 2009; Feldt et al., 2011; Wang et al., 2015).

In AD patients, the deterioration of white matter pathways and the pathological changes in structural connectivity regions are closely related with disruptions in functional connectivity and cognitive decline. For instance, it has been observed that AD patients have increased pathway lengths and decreased global pathway efficiency, impacting information integration and transmission and leading to impaired cognitive and executive functions (Xu et al., 2022). In MCI patients, SC-FC coupling specific to certain networks is significantly reduced, diverging from the correlation with network efficiency (Piao et al., 2023).

Under healthy physiological conditions, β-amyloid (Aβ) is efficiently cleared from the brain through various pathways, including receptor-mediated transport at the blood–brain barrier, enzymatic degradation, and cerebrospinal fluid drainage. However, when there is an imbalance between the production and clearance of Aβ, Aβ gradually accumulates in the central nervous system. Aβ plateaus in later dementia stages despite the continuing functional decline. The production and deposition of Aβ indicate risk of progression to AD (Wang et al., 2021).

The glymphatic system plays a pivotal role in metabolic waste clearance. It forms a neuroglial-dependent perivascular network utilizing the perivascular space (PVS) as a conduit for waste removal. This system is essential not only for managing intracranial metabolic transport and waste removal but also for eliminating interstitial waste from the brain (Iliff et al., 2012). The mechanism involves cerebrospinal fluid being pumped through the subarachnoid space into the periaqueductal area and then transported to the brain parenchyma via aquaporin-4 (AQP4). The interstitial fluid (ISF) and cerebrospinal fluid exchange substances within the loose fibrous matrix along the perivascular space. Once cleared through this perivascular region, interstitial metabolic waste is effectively removed (Xie et al., 2013; Rasmussen et al., 2018).

Recent research has shown that brain regions such as the neocortex and the entorhinal cortex begin to exhibit abnormal protein deposits as early as the MCI stage (Duan et al., 2017; Grothe et al., 2017; Vogels et al., 2020; Berron et al., 2021). Therefore, investigating the mechanisms of abnormal protein production and clearance during the prodromal stage of AD is crucial for deepening our understanding of the early pathological development of the disease, for predicting cognitive decline, and for the staging and early detection of the disease (Jiang et al., 2022; Mahaman et al., 2022). The glymphatic system, by facilitating the exchange of substances between cerebrospinal fluid and interstitial fluid, plays a role in promoting waste clearance. This clearance mechanism is essential for maintaining brain health, as the accumulation of waste can lead to brain damage and increase the risk of neurological diseases. Therefore, glymphatic system dysfunction may be a common and critical phenomenon in the early stages of AD (Nedergaard and Goldman, 2020).

Extensive research, particularly in animal models, has provided a wealth of information regarding the glymphatic system (Harrison et al., 2020; Ishida et al., 2022). These insights have been validated through human studies. Nevertheless, research focusing on glymphatic system function in humans is limited (Eide et al., 2018; Zhou et al., 2020). A significant advancement in this area was the development of a novel noninvasive analytical technique by Taoka and colleagues. This method, known as diffusion tensor image analysis along the perivascular space (DTI-ALPS), is instrumental in assessing the activity of the glymphatic system (Taoka et al., 2017). The high consistency and applicability of DTI-ALPS across various diseases, including AD (Zhang et al., 2023), Parkinson’s disease (PD) (Sundaram et al., 2019), stroke (Toh and Siow, 2021), and traumatic brain injury, have been demonstrated (Sullan et al., 2018). DTI-ALPS serves as a testament to the progress in understanding and evaluating the intricate mechanisms of disease states affecting the brain. Building upon prior work, the complexity of the human brain is reflected in its intricate SC-FC and efficient waste removal mechanisms through the glymphatic system. SC-FC represents the synchronisation between the anatomical pathways of the brain and the functional activity, which is essential for the maintenance of cognitive function. On the other hand, the glymphatic system ensures optimal brain function by removing metabolic wastes. Dysregulation of both can lead to cognitive deficits that characterise neurodegenerative diseases. This study hypothesizes a significant relationship between the brain’s SC-FC coupling and glymphatic system function. Investigating this relationship could provide a deeper understanding into the mechanisms underlying cognitive decline in MCI. To explore this relationship, we simultaneously employed DTI to analyze structural connectivity and fMRI for functional connectivity. The DTI-ALPS index was used to assess glymphatic system activity, to improve the efficacy of diagnostic markers and treatment strategies in MCI.

## 2 Materials and methods

### 2.1 Participants

This study was approved by the Ethics Committee of Guizhou Medical University Affiliated Hospital (Ethics Approval Number 2023 Ethics Review No. (975)), and written informed consent was obtained from all participants or their primary caregivers. From December 2022 to December 2023, we prospectively recruited MCI patients aged 50 to 80 years from the Neuro-Memory Clinic of Guizhou Medical University Affiliated Hospital.

The diagnosis of MCI was made according to the clinical criteria of the National Institute on Aging-Alzheimer’s Association (NIA-AA) (Albert et al., 2011). Patients with secondary dementia, non-Alzheimer’s disease-related MCI, or vascular dementia were excluded. Some patients underwent cerebrospinal fluid or positron emission tomography-computed tomography (PET-CT) for biomarker-based diagnosis. Neurocognitive assessments using the Mini-Mental State Examination (MMSE) were conducted by specialized neurologists. Healthy control participants (HCs) in the study were required to have healthy cognitive function (i.e., and MMSE score of 26–30).

### 2.2 MRI scanning

MRI data were acquired at the Affiliated Hospital of Guizhou Medical University using a 3.0 T Ingenia Elition scanner from Philips Healthcare with a 32-channel head coil. The participants were instructed to lie supine, relax, close their eyes, and remain awake without engaging in active thought to minimize motion and noise artifacts, with cotton balls in their ears and their heads immobilized with foam pads. Three-dimensional T1-weighted structural brain images were captured using a fast field echo sequence with the following parameters: repetition time (TR)/echo time (TE) = 8.1/3.7 ms, field of view (FOV) = 256 mm × 256 mm, matrix size = 256 × 256, flip angle = 8°, and slice thickness = 1 mm, resulting in 176 sagittal slices without gaps. Resting-state fMRI was conducted with a single-shot echo-planar imaging sequence, with TR/TE = 1000/30 ms, FOV = 224 mm × 224 mm, matrix size = 64 × 64, flip angle = 90°, and slice thickness = 3.5 mm, yielding 45 transverse slices and 480 volumes. DTI utilized a similar single-shot EPI sequence, with TR/TE = 3950/96 ms, FOV = 224 mm × 224 mm, matrix size = 112 × 110, flip angle = 90°, slice thickness = 2 mm with no interslice gap, number of signal average (NSA) = 2, 64 diffusion gradient directions (b = 1,000 s/mm^2^) and one volume without diffusion weighting (b = 0 s/mm^2^).

### 2.3 Functional connectivity

Functional MRI data were preprocessed using the DPABI toolkit based on MATLAB (MathWorks, Natick, MA, US, R2021b) software, and the initial 10 time points were discarded to minimize the effects of magnetic field inhomogeneity and acclimatization. The remaining 470 time points were processed using DPARSF software (Yan et al., 2016), which included slice timing correction, head motion correction (excluding data with translation exceeding 3 mm or rotation exceeding 3 degree), normalization (aligning to the Montreal Neurological Institute (MNI) template and resampling to 3 mm^3^ voxels), spatial smoothing (using a 6 mm full-width half maximum (FWHM) Gaussian kernel), detrending, and filtering within the 0.01–0.08 Hz frequency range.

GRETNA software (Wang et al., 2015) was used to construct the whole-brain resting-state functional connectivity network. The brain regions were segmented using the Brainnetome atlas containing 246 subregions of the bilateral hemispheres (BN246), and regions time series were extracted. To address the prevailing disputes and ambiguities, negative correlations within the functional connectivity networks were excluded from this study due to their unclear physiological interpretations.

### 2.4 Structural connectivity

DTI data were processed using the PANDA toolbox (Cui et al., 2013), which involved the following steps. Initially, the raw DTI data were converted to the Neuroimaging Informatics Technology Initiative (NIfTI) format and organized and named according to the PANDA standards. Head motion and eddy current distortions were corrected through affine transformations of the b = 0 images to increase image quality. After brain extraction, nonbrain tissues were removed to increase the accuracy of spatial registration. Fractional anisotropy (FA) was calculated for each voxel. The FA images were then coregistered with the corresponding T1-weighted images using affine transformations in native space. Finally, deterministic fiber tracking, such as the FACT algorithm, was employed to reconstruct white matter tracts, with tracking terminating when streamlines reached voxels with an FA value below 0.2 or when the tracking angle exceeded 45 degrees.

### 2.5 Brain network coupling analysis

**Figure 1:**
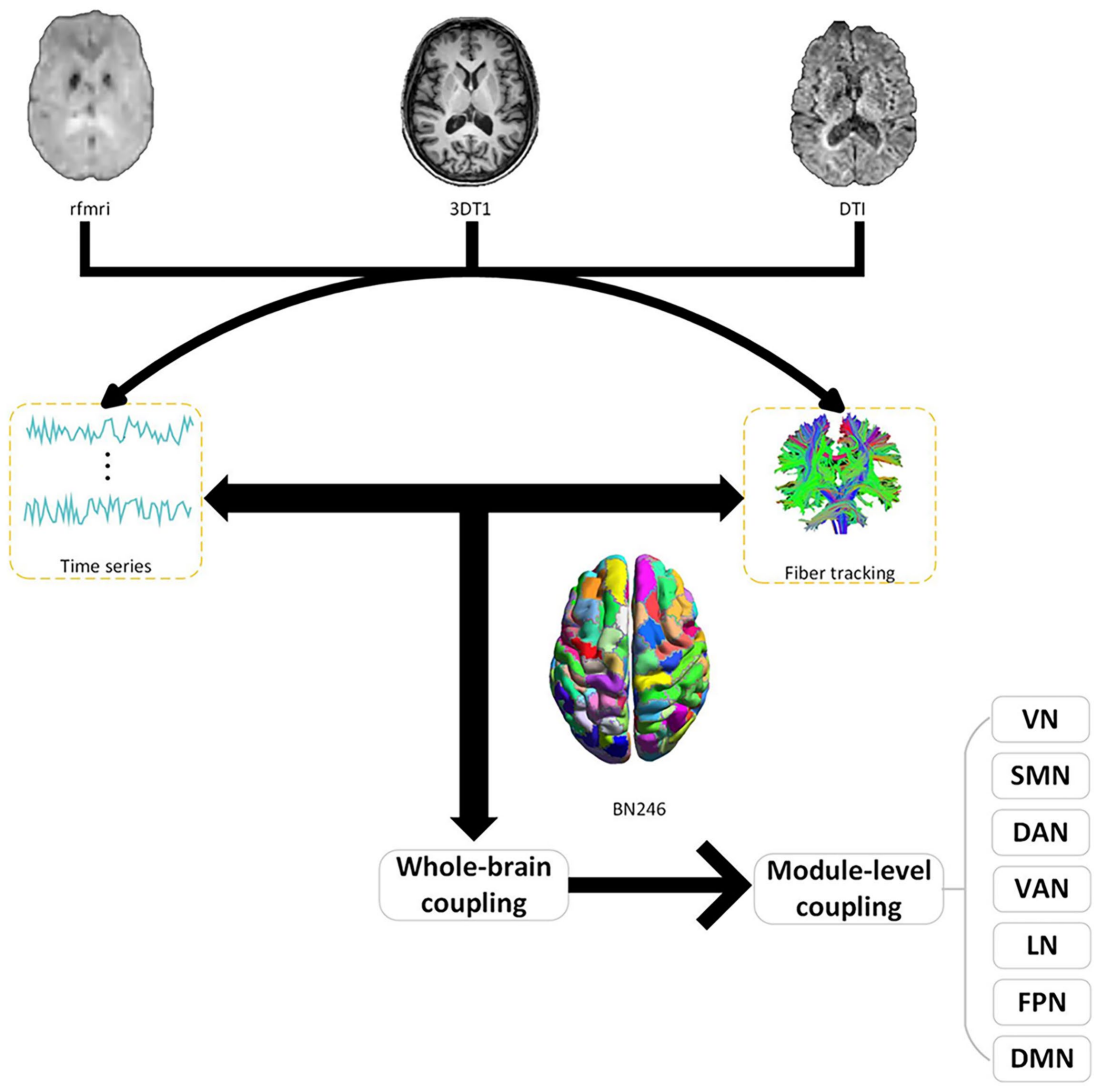
Structure–function coupling flowchart. Initially, a coupling analysis between resting-state functional MRI (fMRI) functional images and diffusion tensor imaging (DTI) structural images was conducted across the whole brain. Subsequently, further coupling analyses were carried out within various subnetworks (VN, visual network; SMN, somatomotor network; DAN, dorsal attention network; VAN, ventral attention; LN, limbic network; FPN, frontoparietal network; DMN, default network). BN246: Brainnetome atlas contains 246 subregions of the bilateral hemispheres.

In our research, we meticulously divided the network nodes into 246 distinct regions of interest (ROIs), a subdivision guided by the Brainnetome Atlas. The BN246 atlas serves as a comprehensive map of the human brain’s network, encompassing 210 regions within the cerebral cortex and an additional 36 in subcortical areas (Fan et al., 2016). We carefully selected 207 brain regions and aligned them with the seven brain networks (visual network, somatomotor network; dorsal attention network; ventral attention; limbic network; frontoparietal network; default network) delineated by Yeo et al. (2011). These regions were then methodically distributed across the seven networks. For a more detailed breakdown of these modules, please refer to Figure 1 and Supplementary Table S1.

### 2.6 ALPS index calculation

For the conversion of 4D DTI volume DICOM files into NIfTI format, we employed the MRIcroGL GUI interface. An in-house bash script was developed to facilitate the computation of the ALPS index, utilizing the DTI images as input and incorporating commands from FSL and MRtrix3. The DTI images were refined to remove artifacts by employing the Marchenko-Pastur Principal Component (MP-PCA) denoising algorithm and Gibbs ringing correction via MRtrix3’s “dwisenoise” and “mrdegibbs” commands. Adjustments for susceptibility-induced distortions, as well as eddy current and movement artifacts, were performed using the “topup” and “eddy” commands in FSL. FA maps, along with diffusivity maps in the x-, y-, and z-axes, were generated using the “dtifit” command in FSL. Each participant’s FA map was aligned to the Johns Hopkins University International Consortium Brain Mapping (JHU-ICBM) FA template, and this transformation matrix was subsequently applied to all diffusivity maps using the “flirt” command in FSL.

**Figure 2:**
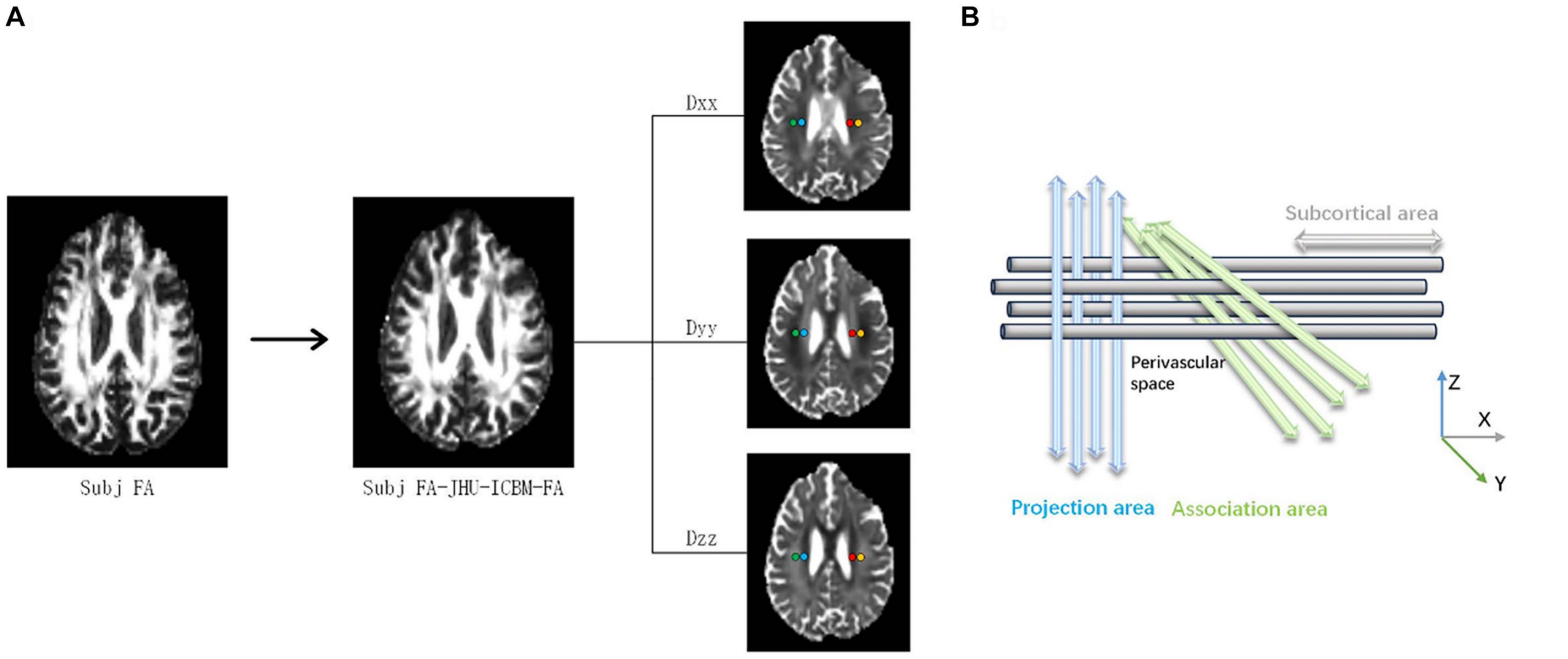
A DTI-ALPS coefficient concept diagram. **(A)** The fractional anisotropy (FA) images of an individual participant are registered to the JHU-ICBM FA template. Color-coded FA maps, along with diffusion maps along the x-axis (right to left; Dxx), y-axis (anterior to posterior; Dyy), and z-axis (inferior to superior; Dzz), were generated. **(B)** The DTI-ALPS coefficient is determined by the ratio of diffusion rates perpendicular to the main fibers. Specifically, the DTI-ALPS coefficient is the ratio of the average diffusion rate along the x-axis in the projection fiber region (Dx_proj) and the association fiber region (Dx_assoc) to the average diffusion rate along the y-axis in the projection fiber region (Dy_proj) and the average diffusion rate along the z-axis in the association fiber region (Dz_assoc).

The superior corona radiata (SCR) and the superior longitudinal fasciculus (SLF) were identified at the level of the lateral ventricle body using the JHU-ICBM-DTI-81 white matter labeled atlas. ROIs were automatically designated as 5 mm diameter spheres in the bilateral SCR and SLF regions, a process consistently applied across all participants’ diffusivity maps. The central coordinates for these ROIs were established as follows: left SCR (116, 110, 99), left SLF (128, 110, 99), right SCR (64, 110, 99), and right SLF (51, 110, 99) based on the JHU-ICBM-FA template. The diffusivity values for Dxx, Dyy, and Dzz in the bilateral SLF and SCR were automatically extracted for ALPS index calculation (Figure 2) (Wang et al., 2023).

### 2.7 Statistics

Statistical analysis was conducted using SPSS 26.0 software (SPSS, IBM Analytics) and R language 4.3.1. Participant characteristics, including sex, age, years of education, and cognitive performance (MMSE scores), were compared between groups. Categorical variables were analyzed using the chi-squared (χ2) test. Analysis was conducted using independent sample *t*-tests for normally distributed data, and Mann–Whitney U tests for non-normally distributed data. Continuous data were reported as Mean (SD) or Median (IQR). For normally distributed data, Pearson correlation analysis was employed, while Spearman correlation analysis was used for skewed data based on the data distribution properties. To predict the condition of MCI patients, we employed the DeLong method to calculate the area under the receiver operating characteristic (ROC) curve, i.e., the Areas under the curve (AUC) value, and we used the Youden index (sensitivity + specificity −1) to determine the cutoff value. The statistical significance threshold was set at *p* < 0.05. Cohen’s d was used to demonstrate the effect size of the results (Kelley and Preacher, 2012).

## 3 Results

### 3.1 Demographic and clinical characteristics of the participants

This study included a total of 41 participants, including 23 MCI patients (67.74 ± 6.99 years of age) and 18 healthy controls (HC) (63.44 ± 6.92 years of age). Table 1 lists the demographic and clinical characteristics of the MCI patients and the HCs. There was no significance between the HC group and the MCI patient group in terms of age, sex, or years of education (*p* = 0.057, 0.326, and 0.095, respectively). Notably, the MMSE scores of the MCI group were significantly lower than those of the HC group [28 (28, 29.75) vs. 21 (16, 23), *p* < 0.001].

**Table 1:** Demographic and clinical data of the participants.

	HC (*n* = 18)	MCI (*n* = 23)	Statistics (*t*/z/χ^2^)	*p* value
Age (years)	63.44 ± 6.92	67.74 ± 6.99	t = 1.961	0.057
Sex (F/M)	9/9	15/8	χ^2^ = 0.963	0.326
Education (years)	9 (7.25, 12)	12 (9, 14)	z = −1.668	0.095
MMSE	28 (28, 29.75)	21 (16, 23)	z = −5.344	<0.001

MMSE, mini-mental state examination.

### 3.2 Decreased SC-FC coupling values in MCI patients

**Figure 3:**
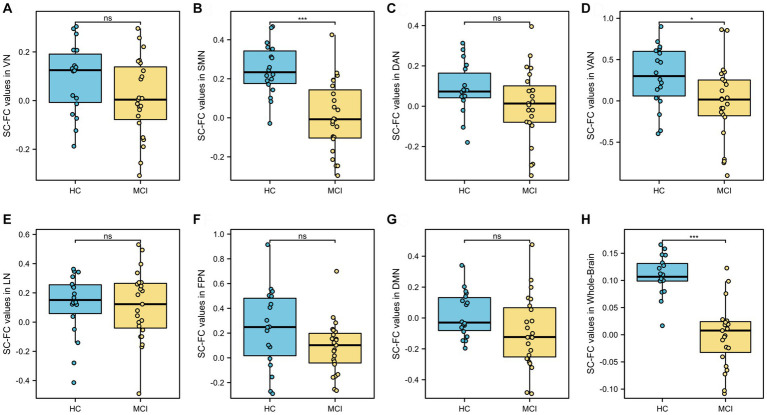
Analysis of differences in whole-brain and seven subnetwork SC-FC coupling values. SC-FC coupling values in SMN, VAN and whole-brain in MCI were lower than those in HC. VN, visual network; SMN, somatomotor network; DAN, dorsal attention network; VAN, ventral attention; LN, limbic network; FPN, frontoparietal network; DMN, default network. ns: insignificant. * *p* < 0.05. ** *p* < 0.01; ***p < 0.001.

At the whole-brain level, SC-FC coupling in MCI patients was significantly lower than that in HCs (*p* < 0.001, *t* = 7.097, Cohen’s d = 2.293), as shown in Figure 3H and Table 2. Among the seven subnetwork modules analyzed, the MCI patients showed lower levels of SC-FC coupling in the SMN (*p* < 0.001, *t* = 4.732, Cohen’s d = 1.519) and ventral attention network (VAN) (*p* = 0.023, *t* = 2.360, Cohen’s d = 0.753) than did the HC group, with very large effect sizes observed for the whole brain and SMN module, as shown in Figures 3B,D,H and Table 2. No significant differences in coupling were detected in VN (*p* = 0.107), DAN (*p* = 0.120), LN (*p* = 0.825), FPN (*p* = 0.072), or DMN (*p* = 0.086), as shown in Figures 3A,C,E–G and Table 2.

**Table 2:** Analysis of differences in the structural-functional coupling values of the whole brain and seven subnetworks.

	HC (*n* = 18)	MCI (*n* = 23)	Statistics (*t*/z)	*p* value	Cohen’s d
VN	0.092 ± 0.143	0.012 ± 0.161	*t* = 1.650	0.107	0.522
SMN	0.247 ± 0.132	0.006 ± 0.182	*t* = 4.732	<0.001	1.519
DAN	0.086 ± 0.126	0.007 ± 0.179	*t* = 1.592	0.120	0.512
VAN	0.293 ± 0.373	−0.023 ± 0.461	*t* = 2.360	0.023	0.753
LN	0.151 (0.058, 0.255)	0.123 (−0.041, 0.265)	z = −0.236	0.813	0.024
FPN	0.238 ± 0.320	0.083 ± 0.213	*t* = 1.853	0.072	0.569
DMN	0.022 ± 0.147	−0.089 ± 0.233	*t* = 1.761	0.086	0.569
Whole-Brain	0.109 ± 0.036	−0.001 ± 0.057	*t* = 7.097	<0.001	2.293

VN, visual network; SMN, somatomotor network; DAN, dorsal attention network; VAN, ventral attention; LN, limbic network; FPN, frontoparietal network; DMN, default network.

**Figure 4:**
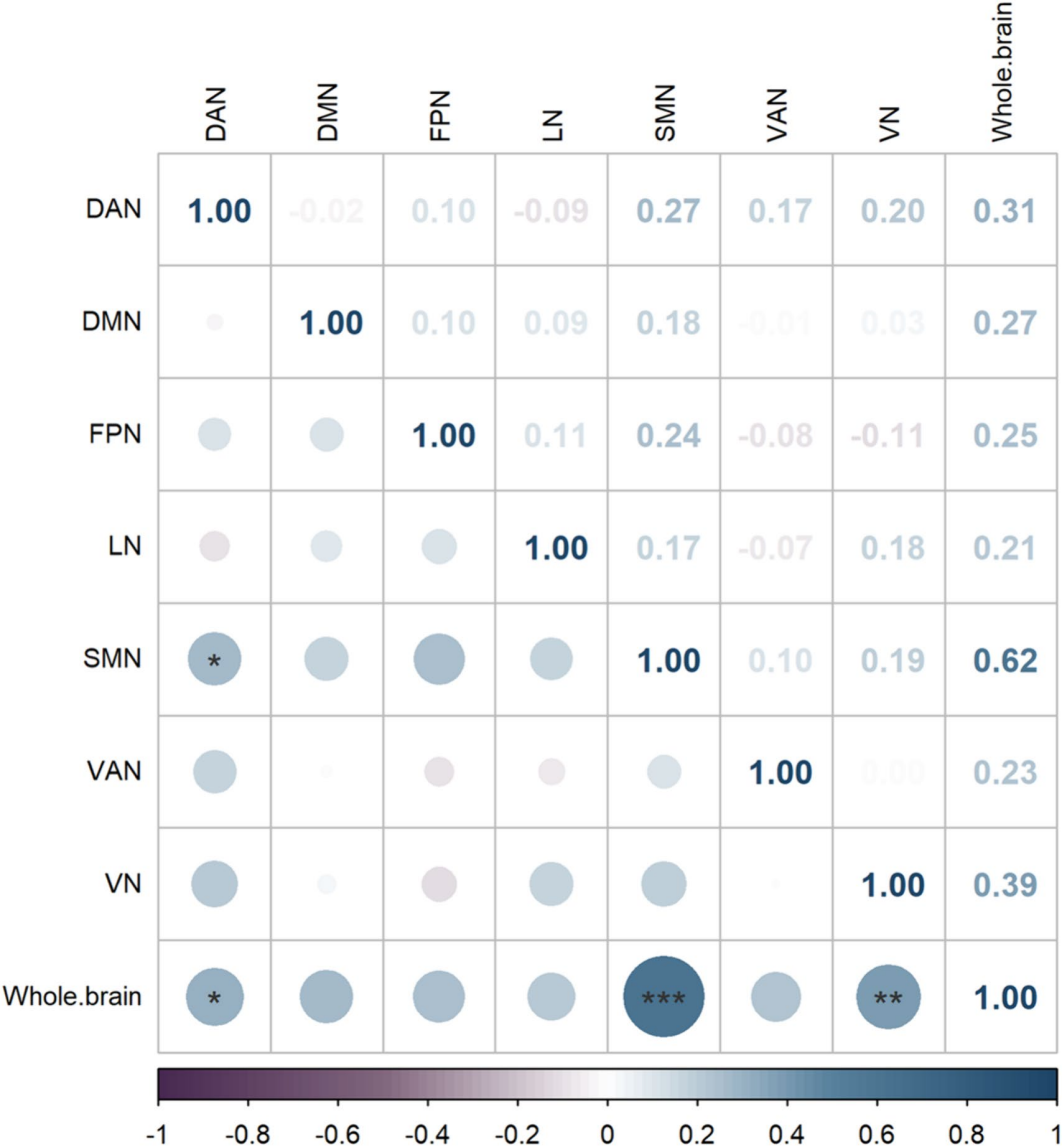
Brain network correlation heatmap, where darker colors indicate higher Spearman correlation coefficient weights.

As shown in Figure 4, the whole-brain network coupling values were significantly positively correlated with the DAN (*p* = 0.045, *r* = 0.314), SMN (*p* < 0.001, *r* = 0.619) and VN (*p* = 0.005, *r* = 0.390). At the module level, there was a significant positive correlation between the DAN module and the SMN module (*p* = 0.039, *r* = 0.266).

### 3.3 Correlation analysis between coupling values and MMSE scores

**Figure 5:**
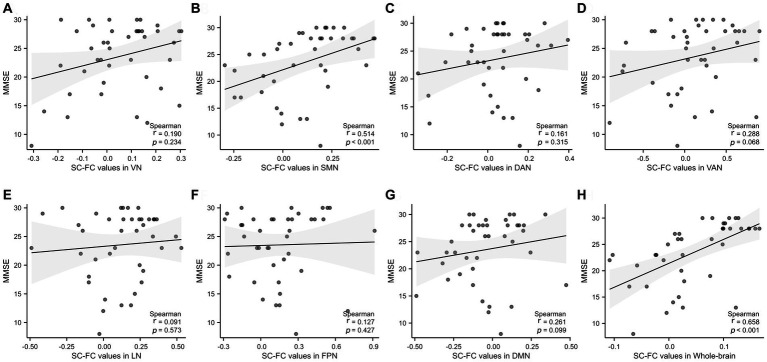
Correlation analysis of the whole brain and 7 subnetworks with MMSE scores. A significant positive correlation was observed between MMSE and SC-FC in the SMN and whole-brain. SMN, somatomotor network; DAN, dorsal attention network; VAN, ventral attention; LN, limbic network; FPN, frontoparietal network; DMN, default network. r: partial correlation coefficient.

There was a significant positive correlation between MMSE scores and the whole-brain network (*p* < 0.001, *r* = 0.658) and the SMN (*p* < 0.001, *r* = 0.514), as shown in Figures 5B,H. No significant correlations were found in VN, DAN, VAN, LN, FPN, DMN (*p* = 0.234, *p* = 0.315, *p* = 0.068, *p* = 0.573, *p* = 0.427, *p* = 0.099, respectively), as indicated in Figures 5A,C–G.

### 3.4 Diagnostic performance comparison of coupling values

**Figure 6:**
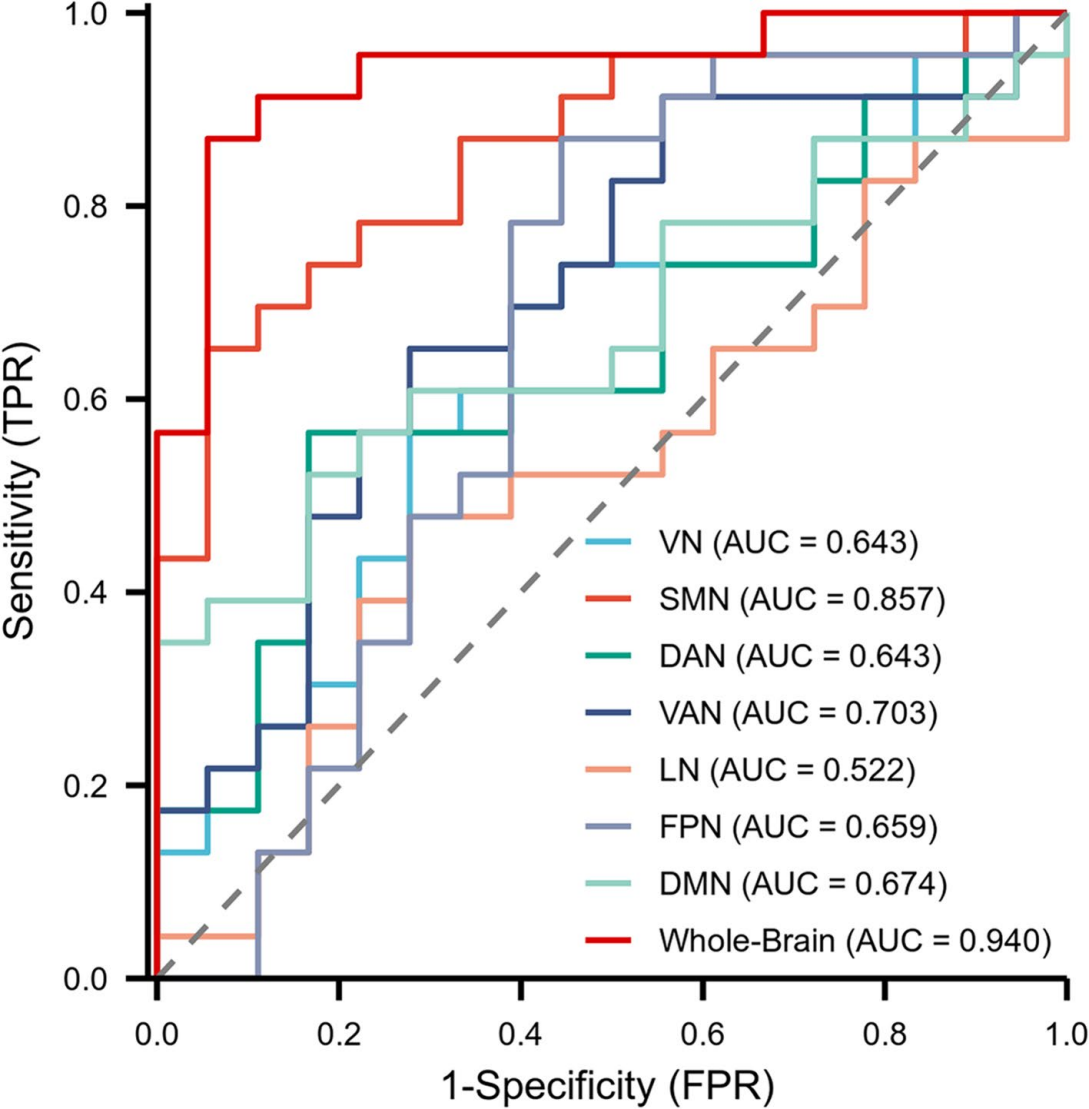
Receiver operating characteristic (ROC) curves showing the diagnostic performance of the whole brain and 7 subnetworks for MCI patients.

The diagnostic capability of whole-brain network coupling was the strongest, with an AUC of 0.940, a sensitivity of 86.96%, a specificity of 94.44%, and a diagnostic threshold of 0.045. SMN coupling (AUC of 0.857, sensitivity of 65.22%, specificity of 94.44%, diagnostic threshold of 0.069) and VAN coupling (AUC of 0.703, sensitivity of 65.22%, specificity of 72.22%, diagnostic threshold of 0.130) had moderate diagnostic performance. The diagnostic efficacy of DAN coupling (AUC of 0.643, sensitivity of 56.52%, specificity of 83.33%, diagnostic threshold of 0.026) and VN coupling (AUC of 0.643, sensitivity of 69.57%, specificity of 61.11%, diagnostic threshold of 0.110) was lower. All the above results were statistically significant (*p* < 0.05). The AUC values of other brain network coupling features were lower but the difference was not significant (Table 3; Figure 6).

**Table 3:** ROC curve parameters for the whole brain and 7 subnetworks.

Predictor variable	Cutoff value	Sensitivity (%)	Specificity (%)	Precision (%)	AUC (95% CI)	*p* value
VN	0.110	69.57%	61.11%	65.85%	0.643 (0.469–0.816)	0.121
SMN	0.069	65.22%	94.44%	78.05%	0.857 (0.743–0.972)	<0.001
DAN	0.026	56.52%	83.33%	68.29%	0.643 (0.469–0.816)	0.121
VAN	0.130	65.22%	72.22%	68.29%	0.703 (0.537–0.868)	0.027
LN	0.099	47.83%	72.22%	58.54%	0.522 (0.339–0.705)	0.813
FPN	0.239	86.96%	55.56%	73.17%	0.659 (0.472–0.846)	0.083
DMN	−0.122	52.17%	83.33%	65.85%	0.674 (0.507–0.841)	0.059
Whole-Brain	0.045	86.96%	94.44%	90.24%	0.940 (0.865–1.000)	<0.001

### 3.5 Analysis of differences in the ALPS index between HCs and individuals with MCI and its relationship with coupling

**Figure 7:**
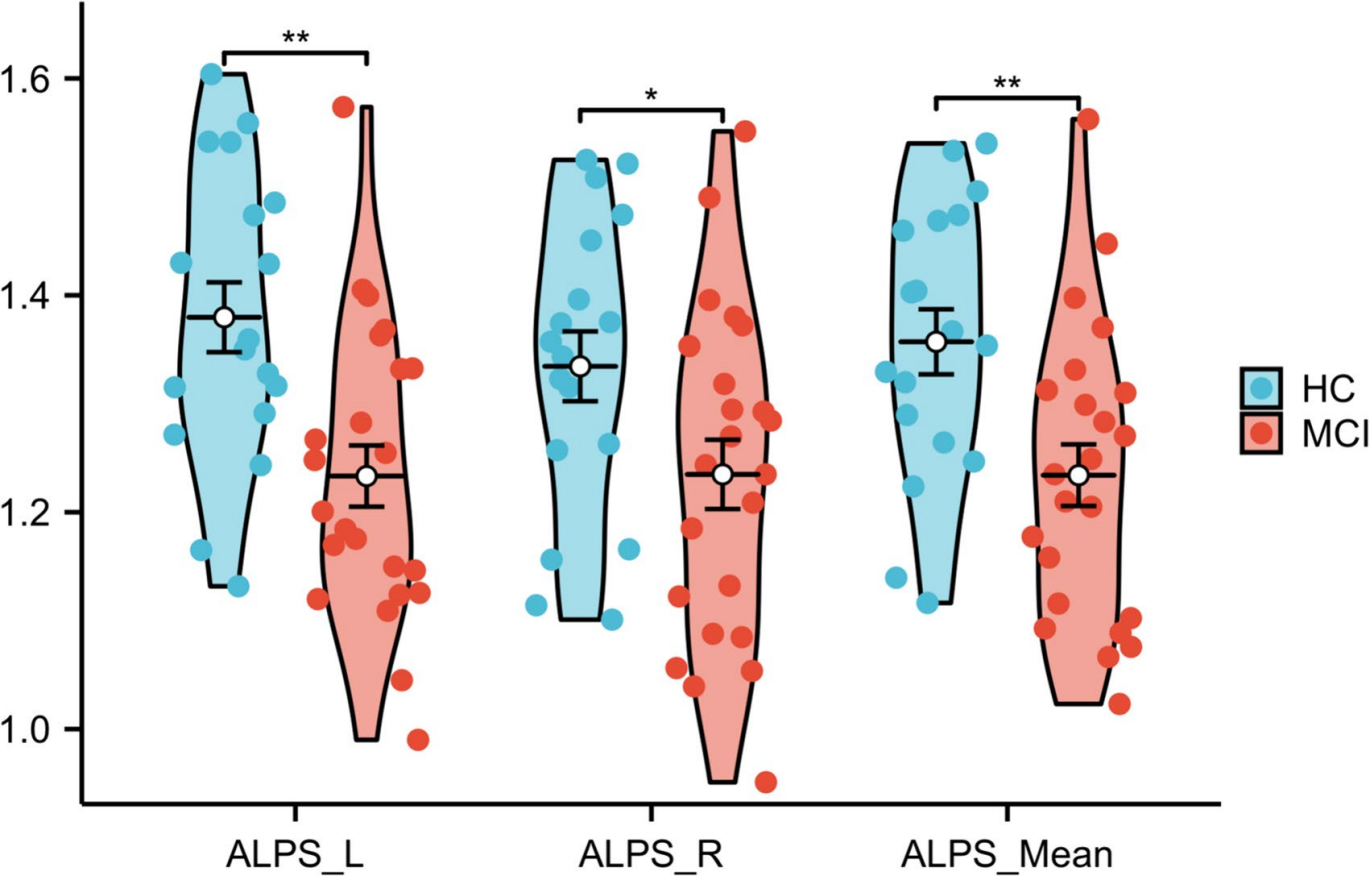
Differences in ALPS_Mean, ALPS_R, and ALPS_L between HCs and MCI patients. ALPS_L: left ALPS index, ALPS_R: right ALPS index, ALPS_Mean: mean of ALPS_L and ALPS_R.

Significant differences were observed between HCs and participants with MCI in the left ALPS index and the right and mean ALPS indices (*p* = 0.001, *t* = 3.422, Cohen’s d = 1.076; *p* = 0.037, *t* = 2.165, Cohen’s d = 0.686; *p* = 0.005, *t* = 2.952, Cohen’s d = 0.933, respectively), with the left ALPS index showing the strongest significance. See Figure 7 and Table 4 for details.

**Table 4:** Differences in ALPS indexes in HCs and participants with MCI.

	HC (*n* = 18)	MCI (*n* = 23)	Statistics (*t*)	*p* value	Cohen’s d
ALPS_L	1.380 ± 0.137	1.233 ± 0.135	3.422	0.001	1.076
ALPS_R	1.334 ± 0.137	1.234 ± 0.153	2.165	0.037	0.686
ALPS_Mean	1.357 ± 0.127	1.234 ± 0.136	2.952	0.005	0.933

ALPS_L: left ALPS index, ALPS_R: right ALPS index, ALPS_Mean: mean of ALPS_L and ALPS_R.

**Figure 8:**
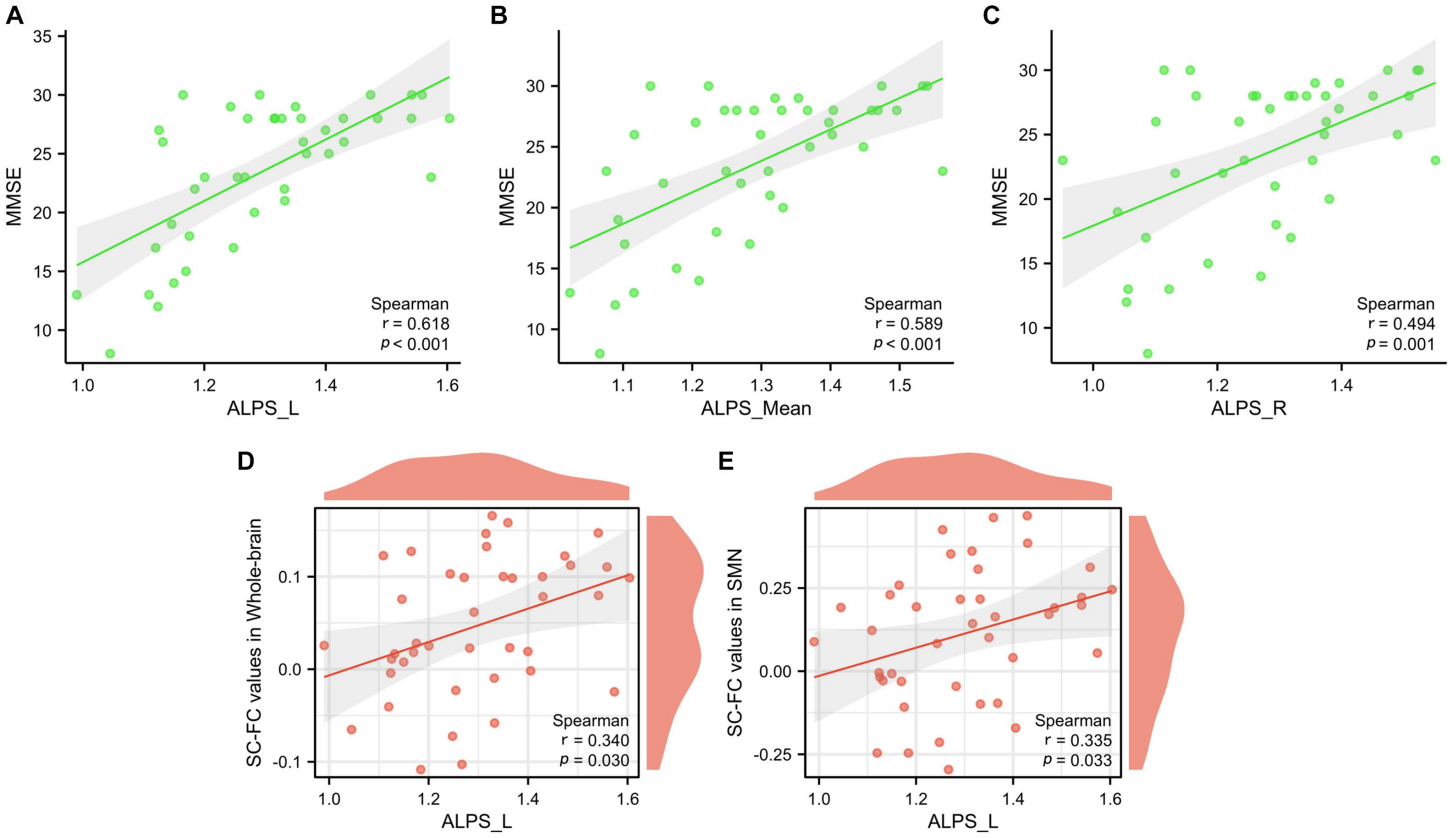
Correlation analysis between glymphatic function and MMSE scores **(A–C)**. Correlation analysis between glymphatic function and whole-brain structural-functional coupling, as well as SMN coupling **(D,E)**.

The left ALPS index (*p* < 0.001, *r* = 0.618), the right ALPS index (*p* < 0.001, *r* = 0.494), and the mean ALPS index (*p* < 0.001, *r* = 0.589) were strongly positively correlated with the MMSE score (Figures 8A–C). Furthermore, a significant correlation was observed between the left ALPS index and whole-brain coupling (*p* = 0.030, *r* = 0.340) (Figure 8D) and SMN coupling (*p* = 0.033, *r* = 0.335) (Figure 8E).

## 4 Discussion

This study represents the first investigation into MCI patients by examining the association between SC-FC and glymphatic system function. The main findings are as follows:

1. Compared to the HCs, MCI patients exhibited a significant weakening of SC-FC coupling at the whole-brain level. At the modular level, MCI patients showed a notable reduction in SC-FC coupling within several key brain network modules, including the VAN and SMN. Besides, there is a notable positive correlation between MMSE scores and both the SC-FC of the whole brain and the SMN.
2. At the level of the whole brain and subnetworks, MCI patients showed significant interactions in SC-FC coupling. In particular, the coupling between the whole brain and the SMN module, as well as between the whole brain and the DAN and VN modules, displayed significant effects. Among the module interactions, the coupling effect between the SMN and DAN modules was the most significant. Furthermore, ROC curve analysis demonstrated that whole-brain network coupling had the best diagnostic performance.
3. The ALPS index of the left hemisphere was significantly correlated with the SC-FC coupling of the whole brain and SMN.

### 4.1 The significance of SC-FC coupling and its application to AD-MCI

Through our study of whole-brain and modular brain networks in patients with MCI, we identified multiscale structural-functional relationships with reduced coupling values of whole-brain, VAN, and SMN in patients with MCI. This finding aligns with earlier research on AD and MCI patients (Xu et al., 2022; Piao et al., 2023), showing significant differences in functional-structural coupling at both the whole-brain and subnetwork levels, with MCI patients exhibiting extensive decoupling phenomena. We found a close association and mutual influence between the whole brain and subnetworks (SMN, DAN and VN), Additionally, correlations with MMSE scores were also found in the SC-FC in whole brain and SMN. This is in line with Esposito et al. (2013) who also found significant differences in SMN functional connectivity between the MCI and HC groups. Dipasquale et al. (2015) further decomposed the SMN by a dimensionality reduction method and found the presence of FC loss in it. These findings suggest that there are deficits in both global and local FC-SC integration in the brains of MCI patients.

Changes in SC-FC coupling also reflect the asynchrony between functional and structural connections. Under healthy conditions, the brain’s functional and structural connections are coordinated and synchronous, jointly maintaining the stability and efficient operation of the brain networks. However, under the influence of neurodegenerative diseases such as AD, this synchrony may be disrupted, leading to changes in SC-FC coupling. The impaired FC and SC networks provide not only crucial imaging markers to help detect and diagnose neurodegenerative diseases such as AD earlier but also important clues for understanding the potential mechanisms behind cognitive deficits in these diseases. By delving deeper into the relationship between changes in SC–FC coupling and neurodegenerative diseases such as AD, we hope to find new therapeutic methods and interventions to improve patients’ quality of life and slow the progression of the disease.

### 4.2 Glymphatic system function and its relationship to SC-FC coupling

In our study, when comparing HCs with MCI patients, MCI patients exhibited a significant decrease in the ALPS index in the left hemisphere, right hemisphere, and on average. This finding suggested a potential decline in glymphatic system function in MCI patients, particularly in the left hemisphere, which is consistent with the findings of Zhong et al. (2023). Furthermore, similar to previous findings, we also discovered a positive correlation between the ALPS index and MMSE score (Taoka et al., 2017; Steward et al., 2021). This correlation indicates that there might be an intrinsic link between the normal functioning of the glymphatic system and cognitive functions and that a decline in glymphatic system function could be an important factor leading to exacerbated cognitive impairments. More interestingly, our study revealed that the significant reduction in glymphatic system function in the left hemisphere is closely related to the structural–functional coupling of the brain. This finding may provide a new perspective for revealing the mechanism of brain network abnormalities in MCI patients. Functional asymmetry particularly refers to the differences between the two hemispheres of the brain in processing various functions. Despite the functional complementarity of the two hemispheres in language tasks, language functions are usually dominated by the left hemisphere, whereas spatial cognition and face recognition are more dependent on the right hemisphere. The theory of the mirror neuron system implies that the relearning process that promotes language fluency and comprehension through action observation may involve organizational changes in both cerebral hemispheres (Oliveira et al., 2017; Olulade et al., 2020). It is also evident in other neural system functions, such as the distribution of glymphatic system functions. Patients with right-sided temporal lobe epilepsy showed more significant impairment of the glymphatic system on the left side, while patients with left-sided temporal lobe epilepsy did not show significant asymmetry (Zhao et al., 2023). Understanding this asymmetry is important for revealing the mechanisms of brain function and its ability to handle complex information.

A recent study, while measuring the dynamics of cerebrospinal fluid flow, captured blood oxygen level-dependent (BOLD) signals using fMRI. The results showed a close correlation between a large influx of cerebrospinal fluid and fluctuations in the BOLD signal. This finding suggests that with the development of new neuroimaging technologies, we are now able to observe both widespread neuronal functional activities and fluid movements of nonneuronal cells simultaneously (Fultz et al., 2019). Research on diffusion fMRI has revealed associations between neural activity and the swelling of astrocytes and neurons (Ferris, 2021), a phenomenon that might occur even without a significant increase in BOLD signals (Abe et al., 2017). Furthermore, higher DTI-ALPS values may be associated with increased diffusivity in the PVS, which could reflect changes in the microstructure of white matter. Conversely, an increased peak width of skeletonized mean diffusivity (PSMD) indicates changes in the microstructure of white matter, reflecting overall white matter damage and potential structural connectivity impairments (Yang et al., 2024). These findings further emphasize the correlation between glymphatic system dysfunction and changes in structural–functional coupling, indicating that cognitive impairment may be the end result of these changes.

Overall, the observed relationship between SC-FC coupling and glymphatic system function underscores the intertwined nature of structural and functional brain networks, revealing how this connection contributes to the development of cognitive and daily living functional impairments in MCI.

### 4.3 Limitations and outlook

Despite its contributions, this study still has some limitations. Conducted at a single center with a relatively small sample size, it may not fully represent the broader patient population, though some findings align with previous research. It focused on static brain connectivity features, overlooking the dynamic nature of FC (Qin et al., 2021; Liu et al., 2022), unlike the relatively stable SC. Additionally, the cross-sectional design limited our ability to track the ALPS index over time. To address these limitations, future research should expand the sample size and adopt a longitudinal approach for more precise data analysis.

## 5 Conclusion

In summary, the findings of this study emphasize the importance of gaining a deep understanding of the changes occurring in brain network connectivity and microstructure in patients with MCI. The results of this study clearly show that these structural and functional changes in the brain have a significant impact on the cognitive functions of patients, as evidenced by the correlation between these changes and MMSE scores. Furthermore, The ALPS index in the left hemisphere was significantly correlated with SC-FC coupling in both the whole brain and the SMN. This suggests that the altered patterns of regional and overall SC-FC coupling, along with the function of the lateralized glymphatic system, may provide new insights into the pathophysiological mechanisms of MCI. This discovery of lateralization suggests that the asymmetric degeneration of brain functions and structures needs to be further explored in future studies to better understand their specific impact on cognitive function impairment, thereby offering new perspectives for the diagnosis and treatment of MCI.

## Data availability statement

The raw data supporting the conclusions of this article will be made available by the authors, without undue reservation.

## Ethics statement

The studies involving humans were approved by Ethics Committee of Guizhou Medical University Affiliated Hospital. The studies were conducted in accordance with the local legislation and institutional requirements. The participants provided their written informed consent to participate in this study.

## Author contributions

Y-WS: Conceptualization, Data curation, Formal analysis, Investigation, Methodology, Resources, Software, Validation, Visualization, Writing – original draft, Writing – review & editing. Xin-YL: Conceptualization, Data curation, Investigation, Methodology, Resources, Software, Validation, Visualization, Writing – review & editing. Xia-YL: Data curation, Investigation, Methodology, Resources, Validation, Writing – review & editing. M-MH: Data curation, Methodology, Project administration, Resources, Supervision, Writing – review & editing, Software. Z-MW: Data curation, Methodology, Resources, Writing – review & editing. BG: Funding acquisition, Supervision, Writing – review & editing.
